# Long-term survival case of a recurrent colon cancer owing to successful resection of a tumor at hepaticojejunostomy: report of a case

**DOI:** 10.1186/s40792-015-0016-6

**Published:** 2015-03-10

**Authors:** Seiji Natsume, Yasuhiro Shimizu, Tsuyoshi Sano, Yoshiki Senda, Seiji Ito, Koji Komori, Tetsuya Abe, Akio Yanagisawa, Kenji Yamao

**Affiliations:** Department of Gastroenterological Surgery, Aichi Cancer Center Hospital, Kanokoden 1-1, Chikusa-ku, Nagoya, Japan; Department of Gastroenterological Surgery, Aichi Medical University Hospital, 1-1 Yazakokarimata, Nagakute, Aichi Japan; Department of Surgical Pathology, Kyoto Prefectural University of Medicine, 465 Kajiimachi, Hirokoji-noboru, Kawaramachi-dori, Kamikyo-ku, Kyoto Japan; Department of Gastroenterology, Aichi Cancer Center Hospital, Kanokoden 1-1, Chikusa-ku, Nagoya, Japan

**Keywords:** Recurrent colon cancer, Anastomotic recurrence, Intramural recurrence

## Abstract

With advances in surgical procedures and perioperative management, hepato-biliary-pancreatic surgery, including hepatectomy and pancreaticoduodenectomy, has been employed for recurrent colon cancer. However, no report has described a case of major hepatectomy with the combined resection of hepaticojejunostomy following pancreaticoduodenectomy for locoregionally recurrent colon cancer. Here, such a case is reported. The patient, a 37-year-old woman, had undergone pancreaticoduodenectomy for lymph node recurrence along the extrahepatic bile duct from cecal cancer. Thirteen months later, a biliary stricture was found at the hepaticojejunostomy site and right hepatectomy was performed. The resected specimen showed a papillary tumor at the hepaticojejunostomy. Based on its histological features, the pathogenesis of this tumor was considered to be intramural recurrence via lymphatic vessels. Although she underwent resection of a lymph node recurrence at her mesentery 12 months later, she has remained well thereafter, without any sign of further recurrence during 5 years of follow-up after hepatectomy.

## Background

The management of locoregionally recurrent colon cancer poses a surgical challenge since these lesions often extend into surrounding structures and organs. Surgery is the sole curative modality that can result in long-term survival after complete resection alone [1]. Usually, the abdominal wall, ureter, kidney, stomach, uterus, or distal part of the pancreas is resected in attempts to achieve complete resection. In contrast, pancreaticoduodenectomy (PD) and major hepatectomy with combined resection of the extrahepatic bile duct are rarely indicated. Herein, we report a case in which a tumor at the site of hepaticojejunostomy was successfully resected with a right hemihepatectomy 2 years after PD for lymph node recurrence of cecal cancer.

## Case presentation

**Figure 1:**
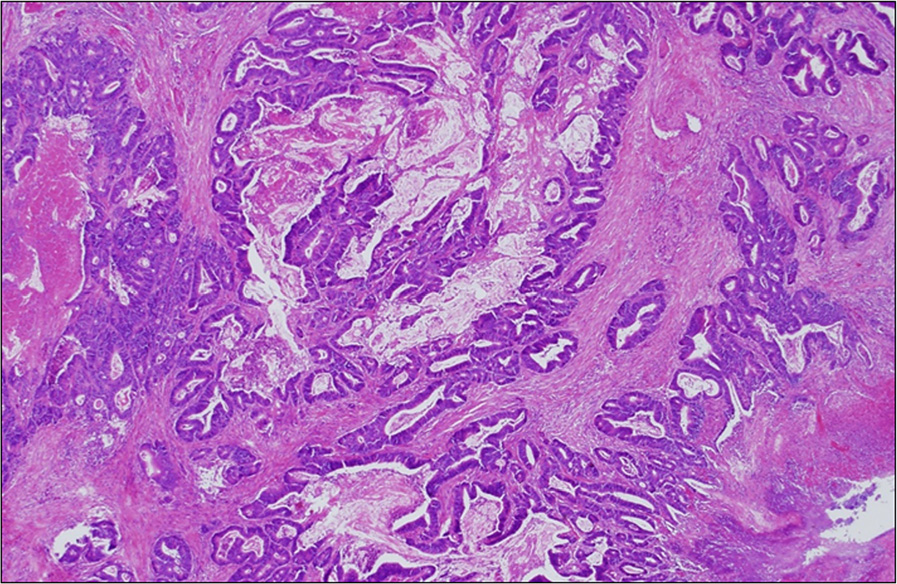
**Histological findings of the initial operation.** Histological examination of initial operation revealed a well-differentiated adenocarcinoma with mucinous carcinoma (H&E, hematoxylin and eosin stain, low magnification).

**Figure 2:**
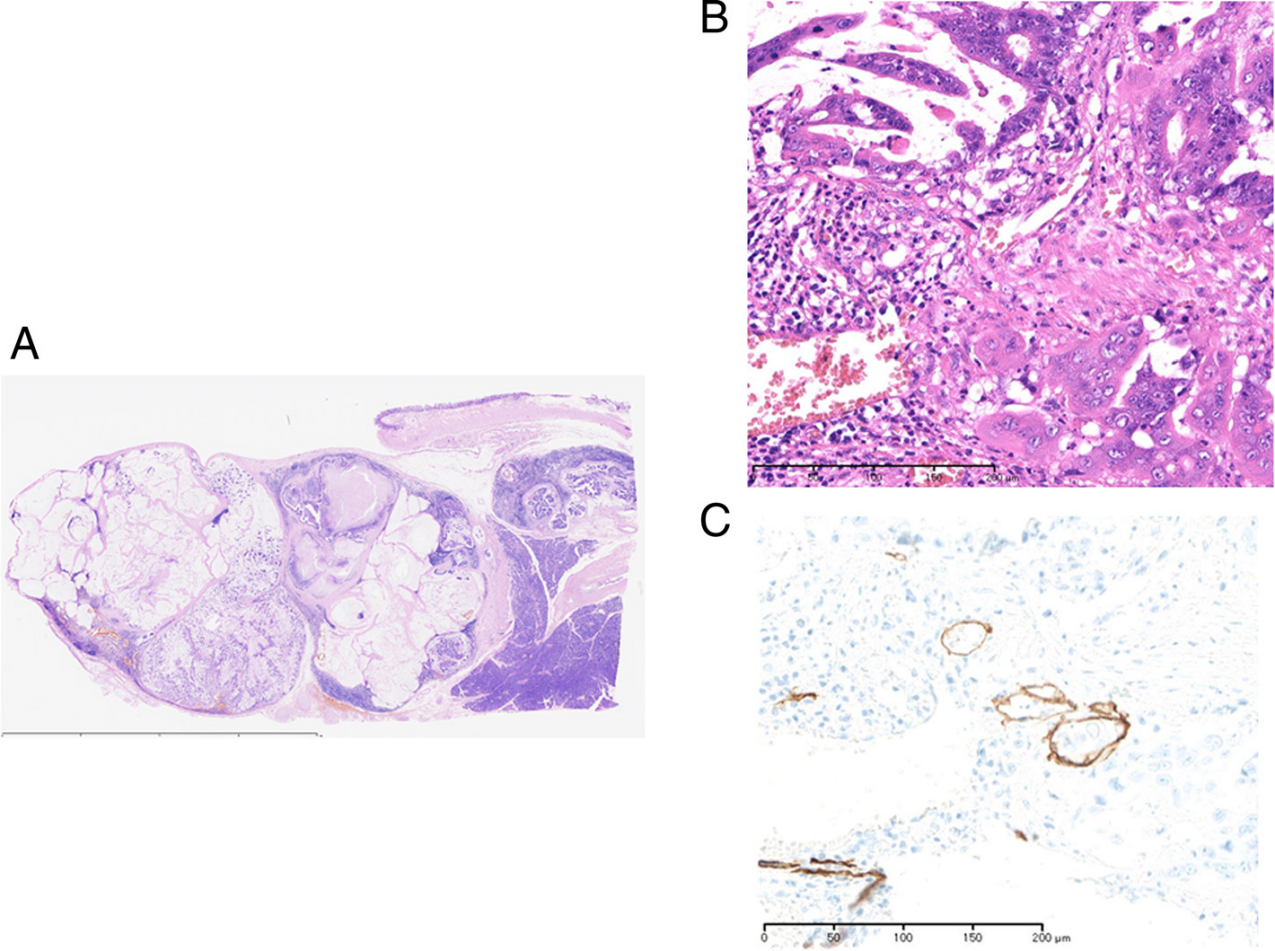
**Histological findings of the second operation.** Histologically, the lymph node along the extrahepatic bile duct was involved by well-differentiated adenocarcinoma with mucinous carcinoma, which is similar to cecal cancer (**(A)** H&E, original magnification; **(B)** H&E, low magnification). Immunostaining with D2-40 monoclonal antibody which is a specific marker for lymphatic vessel elucidated tumor invasion to lymphatic vessels **(C)**.

The patient was a 37-year-old woman who had undergone right hemicolectomy for cecal cancer at a different hospital. Histological examination revealed a well-differentiated adenocarcinoma with mucinous carcinoma, which had invaded the serosal layer without regional lymph node metastasis. A slight lymphatic and vascular invasion was evident. Finally, her tumor was diagnosed as T4aN0M0, stage II B according to UICC classification [2] (Figure 1). Adjuvant chemotherapy with tegafur-uracil and leucovorin (UFT/UZEL) was administered. However, 6 months after her initial operation, a tumor along the extrahepatic bile duct (diameter: 2 cm) was detected by computed tomography (CT). Although hyperbilirubinemia or elevation of biliary enzyme was not seen in her laboratory study, the tumor was located extremely close to the dorsal part of the bile duct and pancreatic parenchyma. It was considered to be a lymph node metastasis from cecal cancer. First, she was treated with systemic chemotherapy of oxaliplatin with fluorouracil and folinic acid (FOLFOX4) for nine courses and irinotecan with fluorouracil and folinic acid (FOLFILI) for six courses. However, chemotherapy was not effective for her recurrent tumor. Moreover, she suffered from severe adverse effect of chemotherapy including grade 3 diarrhea, peripheral neuropathy, and grade 2 neutropenia and could not walk by herself. Thus, she was referred to our hospital for surgical treatment. Since major vessel involvement of the recurrent tumor was not shown by CT, we considered that complete resection would be achieved by pancreaticoduodenectomy. She underwent subtotal stomach-preserving pancreaticoduodenectomy. Histologically, the lymph node along the extrahepatic bile duct was involved by well-differentiated adenocarcinoma with mucinous carcinoma, which is similar to cecal cancer. Tumor invasion to the lymphatic vessels was also observed (Figure 2A,B,C).

**Figure 3:**
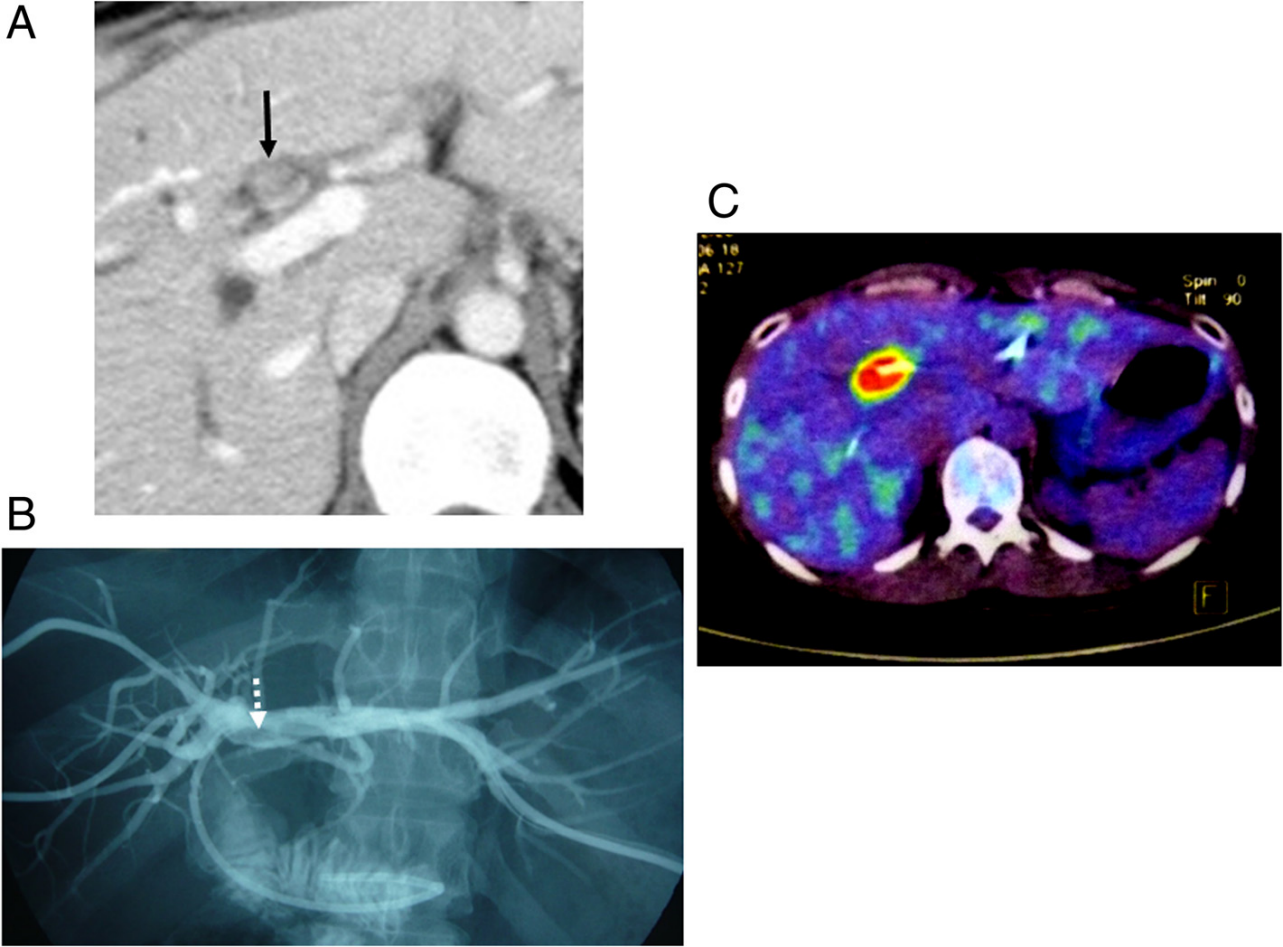
**Preoperative images of the third operation. (A)** Computed tomography demonstrated wall thickening with mild enhancement of the bile duct at the hepatic bifurcation adjacent to the jejunum (arrow) and upstream dilatation of the bilateral hepatic ducts. **(B)** Catheter cholangiogram through the percutaneous transhepatic biliary drainage tube revealed a filling defect at the site of hepaticojejunostomy (dotted arrow). **(C)** Positron-emission tomography revealed an abnormal uptake at the anastomotic site.

**Figure 4:**
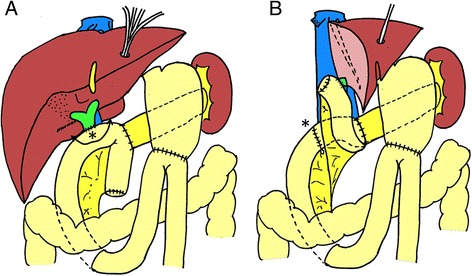
**Schematic presentation of the operative procedures. (A)** The jejunum around the anastomosis (*) was resected with a 1-cm margin (arrow line). **(B)** The jejunum was closed with suturing transversally (*). A renewed hepaticojejunostomy was created between the left hepatic duct and the inverted blind end of the jejunum.

**Figure 5:**
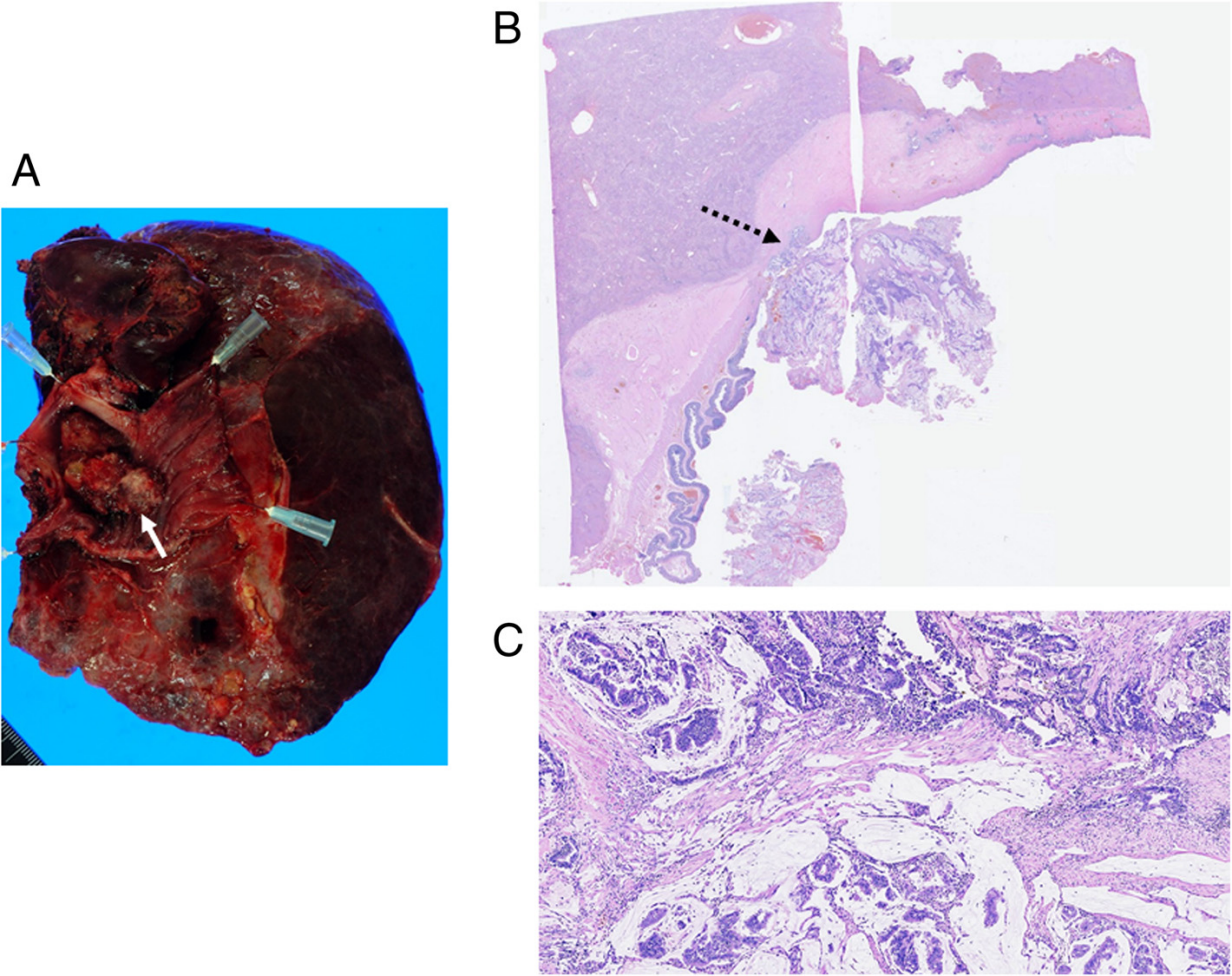
**The resected specimen and microscopic findings. (A)** The resected specimen contained a polypoid tumor at the hepaticojejunostomy (arrow). **(B, C)** Microscopic findings showed well-differentiated adenocarcinoma with mucinous carcinoma similar to the primary and secondary cancers. The polypoid tumor grew from the submucosal layer of the bile duct (dotted arrow), in which many dilated lymphatic vessels with tumor invasion were observed. Intraepithelial spread of tumor cells was evident in neither the bile duct nor the jejunum (**(B)** H&E, original magnification; **(C)** H&E, high magnification).

For 13 months after her second operation, the patient remained well without any adjuvant therapy. However, she subsequently manifested obstructive jaundice. CT demonstrated wall thickening with mild enhancement of the bile duct at the hepatic bifurcation adjacent to the jejunum and upstream dilatation of the bilateral hepatic ducts (Figure 3A). Cholangiography via a percutaneous transhepatic biliary drainage tube revealed a filling defect at the hepaticojejunostomy (Figure 3B). Because a biopsy specimen taken via percutaneous transhepatic cholangioscopy did not show malignant cells, she did not receive further treatment, with the exception of biliary stenting. However, cholangiography performed 8 months later revealed an enlarged filling defect extending from hepatic bifurcation to the confluence of the right anterior and posterior branch. Moreover, positron-emission tomography revealed an abnormal uptake at the anastomotic site (Figure 3C). From these, she was diagnosed as having anastomotic recurrence. Although we proposed an alternative plan of neoadjuvant chemotherapy, she strongly refused it for fear of severe adverse effect which she had experienced after initial recurrence. Moreover, major vessel involvement of the anastomotic tumor was not shown by CT. Thus, we considered that complete resection of the tumor would be achieved by hepatectomy with bile duct resection. She underwent a third operation 2 years after her second operation. Radical surgery was performed by *en bloc* resection of the right hepatic and caudate lobes and the anastomotic region of the bile duct and jejunum. The jejunum around the anastomosis was resected with a 1-cm margin and closed by suturing transversally (Figure 4A). A renewed hepaticojejunostomy was created between the left hepatic duct and the inverted blind end of the jejunum (Figure 4B). The operation time was 413 min and there was 690 ml of blood loss. The resected specimen contained a polypoid tumor at the hepaticojejunostomy (Figure 5A). Microscopic findings showed well-differentiated adenocarcinoma with mucinous carcinoma similar to the primary and secondary cancers. The polypoid tumor grew from the submucosal layer of the bile duct, in which many dilated lymphatic vessels with tumor invasion were observed. Intraepithelial spread of tumor cells was evident in neither the bile duct nor the jejunum (Figure 5B,C).

The patient was discharged from the hospital 26 days after the operation without any complications. Although she underwent a fourth operation for lymph node recurrence at her mesentery 12 months later, she has remained well thereafter, without any sign of further recurrence during the 5 years of follow-up after the hepatectomy.

## Discussion

Distant sites, such as the liver or lung, account for the majority of initial recurrence of resected colon cancers. Locoregional recurrence, as the first site of relapse, is much less common, constituting 10% to 20% of all recurrences [3,4]. Surgery is the only potentially curative therapy for patients suffering from locoregionally recurrent colon cancer. Outcomes following salvage surgery are closely associated with the completeness of resection. It has been reported that patients who underwent curative resection had a 5-year survival rate of 58% and a median survival time of 66 months, while no patients in incomplete resection cohorts survived 5 years [5]. Therefore, surgeons should perform surgery if complete resection will be promising. However, needless to say, the indication for surgical treatment should be carefully determined with consideration of risks and benefits of surgery. In addition, neoadjuvant multidisciplinary approach including chemotherapy or molecular targeted therapy has a possibility of contributing to achieve complete resection and long-term survival. In our case, since the surgical stress of pancreaticoduodenectomy or hepatectomy with bile duct resection is extremely large and incomplete resection might shorten the duration of survival, we carefully discussed the treatment strategy before the second and third operation. However, unfortunately, the adverse effect of chemotherapy was severe, and it was not effective for her recurrent tumor.

With advances in hepato-biliary-pancreatic surgery, hepatopancreaticoduodenectomy (HPD) has been employed for advanced biliary tract cancer with relatively low morbidity and mortality [6,7]. Moreover, a case in which HPD was performed for recurrent or metastatic cancer has also been reported [8,9]. Few reports have described cases of major hepatectomy with combined resection of hepaticojejunostomy following PD, although several reports have described cases of simple hepatectomy following PD without resection of bilioenteric anastomosis [10,11]. This may be explained by the technical difficulties and the extremely rarity of performing two major operations in a single patient at different times. To the best of our knowledge, the English language literature includes only seven cases of major hepatectomy with combined resection of hepaticojejunostomy following PD, including our own case (Table 1) [12-15]. The patient was male in four cases and female in three cases. The average age of the patient was 59.9 years. In six cases, the primary disease was bile duct cancer; the primary disease was recurrent colon cancer in our case alone. The average time between the two operations was 68.3 months. In all cases, right hepatectomy was selected. Secondary surgery was performed for recurrence of the primary lesion in four cases (including ours), while it was performed for a second primary cancer in the remaining three cases. There were two cases in which the patient survived longer than 3 years.

**Table 1:** **Reported seven cases of patients undergoing major hepatectomy with combined resection of hepaticojejunostomy following PD**

**No**	**Authors**	**Year**	**Age**	**Sex**	**Primary disease**	**Time (M)**	**Type of hepatectomy**	**Pathogenesis of secondary lesion**	**Outcome**
1	Seki	1998	75	M	Bile duct Ca	50	Right hepatectomy	Second primary cancer	6 M, D
2	Seki	1998	68	M	Bile duct Ca	153	Right hepatectomy	Second primary cancer	6 M, D
3	Seki	1998	69	F	Bile duct Ca	70	Right hepatectomy	Recurrence	17 M, A
4	Hibi	2006	65	M	Bile duct Ca	36	Right hepatectomy	Second primary cancer	8 M, A
5	Sasaki	2006	45	M	Bile duct Ca	108	Right hepatectomy	Recurrence	30 M, D
6	Okamura	2011	60	F	Bile duct Ca	48	Right hepatectomy	Recurrence	40 M, A
7	Current	2014	37	F	Cecal Ca	13	Right hepatectomy	Recurrence	64 M, A

PD, pancreaticoduodenectomy; No, number of patients; Time (M), time between two operations (months); Outcome, prognosis after the second surgery; M, month; D, dead; A, alive; Ca, cancer.

In the current case, the anastomotic tumor may have been the result of intramural recurrence via the lymphatic vessels because tumor invasion to the lymphatic vessels was observed in both the metastasized lymph node that was resected with PD and the anastomotic tumor (microscopically). We considered two other possible explanations of the pathogenesis. The first relates to the development of the second primary cholangiocarcinoma after bilioenteric anastomosis. Repeated chronic inflammation due to regurgitation from anastomosis evokes epithelial damage, resulting in carcinogenesis, and is the only independent predictor of the development of cholangiocarcinoma [16]. However, the pathological features of the anastomotic tumor were inconsistent with this etiology because findings that suggest chronic inflammatory reaction were absent and intraepithelial tumor cells were not seen in the bile duct mucosa, except for the anastomotic lesion. The second possible explanation is intraluminal implantation. This is a well-known manner of recurrence in colorectal cancer. Gertsch et al. [17] showed that, after completing anastomosis with a circular stapler, malignant cells were collected in the centrifuged saline in which the stapler had been washed. This type of recurrence has also been reported in bile duct carcinoma. Tanaka et al. [18] reported a case of anastomotic recurrence at hepaticojejunostomy in a 10-year survivor of bile duct carcinoma and demonstrated that possible mechanisms of recurrence were a suture implantation or the deposition of intraluminal tumor cells at the anastomotic site. In our case, this is less likely to be the etiology of the anastomotic tumor because tumor cells must be absent from the bile duct when performing hepaticojejunostomy in the previous operation. Nonetheless, the possibility of implantation via the surgical forceps that were used during dissection of the metastatic lymph nodes could not be completely denied.

In the present case, a renewed hepaticojejunostomy was created between the left hepatic duct and the inverted blind end of the jejunum. However, in all previously reported cases, bilioenteric continuity was reestablished using the lower jejunum. At the previous surgery, the lifted jejunum and its mesentery (for bilio- and pancreato-enteric reconstruction) were usually fixed to the mesentery of the transverse colon in order to prevent internal hernia. Therefore, the lower jejunum was unlikely to have enough length to complete the renewed hepaticojejunostomy safely. Our method appears to be advantageous when the blind end of the jejunum is sufficiently long.

## Conclusions

We have reported a case of a tumor at hepaticojejunostomy that was successfully resected with right hemihepatectomy 2 years after pancreaticoduodenectomy for lymph node recurrence of cecal cancer. The pathogenesis of the anastomotic tumor was considered to be intramural recurrence via lymphatic vessels. Aggressive surgery should be performed to achieve long-term survival in cases of locoregionally recurrent colon cancer, even if the surgery is technically demanding.

## Consent

Written informed consent was obtained from the patient for publication of this case report and any accompanying images. A copy of the written consent is available for review by the Editor-in-Chief of this journal.

## Abbreviations

CT: computed tomography
HPD: hepatopancreaticoduodenectomy
PD: pancreaticoduodenectomy
